# Comparison of the Efficacies of Loop-Mediated Isothermal Amplification, Fluorescence Smear Microscopy and Culture for the Diagnosis of Tuberculosis

**DOI:** 10.1371/journal.pone.0021007

**Published:** 2011-06-17

**Authors:** Geojith George, Prem Mony, John Kenneth

**Affiliations:** 1 Division of Infectious Disease, St. John's Research Institute, Bangalore, India; 2 Division of Epidemiology and Biostatistics, St. John's Research Institute, Bangalore, India; New England Biolabs, Inc., United States of America

## Abstract

**Background:**

Despite the advent of novel diagnostic techniques, smear microscopy remains as the most practical test available in resource-limited settings for tuberculosis (TB) diagnosis. Due to the low sensitivity of microscopy and the long time required for culture, feasible and accessible rapid diagnostic methods are urgently needed. Loop-mediated Isothermal Amplification (LAMP) is a promising nucleic-acid amplification assay, which could be accessible, cost-effective and more suited for use with unpurified samples.

**Methodology/Principal Findings:**

In the current study, the objective was to assess the efficacy of a LAMP assay for tuberculosis compared with fluorescence smear microscopy as well as Löwenstein-Jensen (LJ) and Mycobacteria Growth Indicator Tube (MGIT) cultures for the diagnosis of pulmonary tuberculosis using sputum samples. Smear microscopy and culture were performed for decontaminated and concentrated sputum from TB suspects and the LAMP was also performed on these specimens. The LAMP and smear microscopy were compared, in series and in parallel, to culture. LAMP and smear microscopy showed sensitivities of 79.5% and 82.1% respectively and specificities of 93.8% and 96.9% respectively, compared to culture. LAMP and smear in series had sensitivity and specificity of 79.5% and 100.0% respectively. LAMP and smear in parallel had sensitivity and specificity of 82.1% and 90.6% respectively.

**Conclusions/Significance:**

The overall efficacies of LAMP and fluorescence smear microscopy in the current study were high and broadly similar. LAMP and smear in series had high specificity (100.0%) and can be used as a rule-in test combination. However, the performance of LAMP in smear negative samples was found to be insufficient.

## Introduction

Tuberculosis (TB) is one of the oldest diseases that still afflict mankind. The dual specters of TB and AIDS have drawn recent attention to the lack of a suitable diagnostics for TB [1], [2], [3], [4]. TB case detection is the first hurdle towards tackling the TB epidemic [5]. However, the culture which is considered as the ‘gold standard’ of TB diagnosis takes 3–6 weeks, leaving the less sensitive smear microscopy as the only feasible rapid test presently. Even the automated liquid culture systems like BACTEC or Mycobacteria Growth Indicator Tube (MGIT) take 1–6 weeks for growth detection. The utility of microscopy decreases radically in paucibacillary and HIV positive TB suspects. Smear negative carriers, even if considered less infectious, can still spread TB [6]. The long delays in diagnosis result in patients dropping out or continuing to spread TB till they are correctly diagnosed, found and treated [7], [8]. While treating all suspected cases adds significantly to the cost of TB control programs, it also exposes subjects to unnecessary drugs and worsens the emergence of drug resistance. A highly sensitive rule-in test can significantly improve the case detection whereas a highly specific rule-out test can reduce the turnaround time and the duration of respiratory isolation as well as avoid unnecessary administration of potentially toxic drugs [9], [10], [11].

Despite the recent advances in TB diagnosis [12], cost and accessibility continue to be the major limiting factors in the effort to eradicate tuberculosis [13]. Notwithstanding the advent of novel diagnostic techniques, smear microscopy remains the most practical test available in resource-limited settings, where majority of the TB is present. Considerable effort and resources have been invested in developing novel diagnostics and improving existing ones [5], [14], [15]. However, the improvements in sensitivity and specificity achieved thus far have not been extensively demonstrated or rigorously evaluated with actual patient samples in field conditions. Nucleic-acid amplification based tests (NAATs) are of particular interest, since they may be eminently suited for use with respiratory specimens [16] and due to their rapidity and specificity, especially compared to serological tests [17], [18], [19]. Nucleic acid assays are also more amenable to miniaturization and microfabrication, opening new vistas for cost reduction and automation [20]. Loop-mediated Isothermal Amplification (LAMP) was shown to be a promising nucleic-acid amplification assay, which could be accessible and cost-effective [21]. It could also be more robust than other nucleic acid amplification tests, retaining the specificity across wider pH and temperature gradients and showing lesser inhibition in unpurified samples [22]. Suitability of LAMP as a point of care test for the diagnosis of pulmonary tuberculosis is beginning to be evaluated with clinical samples [23], [24].

The LAMP assay was found to be suitable for the laboratory identification of *M. tuberculosis* (MTB) in culture isolates by the authors previously [25]. In the current study, the objective was to assess the efficacy of a LAMP assay for tuberculosis, alone and in combination with fluorescence smear microscopy as well as Löwenstein-Jensen (LJ) and Mycobacteria Growth Indicator Tube (MGIT) cultures for the detection of *M. tuberculosis* from archived sputum samples.

## Materials and Methods

### Ethics Statement

This study was reviewed and cleared by the St. John's Medical College Hospital ethics review board. Written informed consent was obtained from all the participants. The data were analyzed anonymously.

### Participants

To compare ‘LAMP’ with the fluorescence smear microscopy and LJ culture, we used 78 sputum samples obtained from as many TB suspects from the Palamaner region in the state of Andhra Pradesh in southern India.

### Procedures

Samples were collected over a period of six months starting from January 2007. These were decontaminated using NALC-NaOH method and stored at −20°C. Sputum sample digestion-decontamination, LJ culture [26] and auramine O fluorescence microscopy were performed as per standard literature [27]. MGIT cultures were performed as per the manufacturer's instructions. Decontaminated sputum was processed (January to February 2010) using the ‘AMPLICOR Respiratory Specimen Preparation Kit’ (Roche Diagnostics GmbH, Mannheim), according to manufacturer's instructions and the resulting lysate was stored at −20°C. LJ and MGIT cultures were used as the ‘gold standard’ against which other tests could be assessed. The tests were executed and read by experienced personnel, who were blinded to the results of the other tests.

### LAMP reaction

The *M. tuberculosis* specific LAMP reaction was carried out as published previously [28], but was modified to suit local conditions [25]. Briefly, the primers, along with 5 µl of the sample lysate were heat denatured for 3 minutes and then annealed prior to adding the enzyme-dNTP mix. The LAMP reaction was carried out in a final volume of 25 µl in a 120 minute format and was terminated by heating at 80°C for 2 minutes. This assay is specific for the *rimM* sequence of *M. tuberculosis* and *M. bovis*. DNA extracted from *M. tuberculosis* ATCC strain H37Rv was used as the positive control and PCR grade water as the negative control. The *Bst* polymerase (Large fragment) was purchased from New England Biolabs Inc. The primers and SYBR Green I were acquired from Sigma-Aldrich Corporation, India. The final reaction volume was 25 µl (including sample volume 5 µl).The results were visualized by adding 2 µl of 10-fold diluted original SYBR Green I after amplification, and confirmed by agarose gel electrophoresis [29].

### Statistical methods

Data was analyzed using SPSS software - version 17 (© IBM corporation 2010). Sensitivity, specificity [30] as well as positive and negative predictive values (PPV & NPV) [31] were calculated for the LAMP and smear microscopy in comparison with culture as the gold standard. LAMP and smear microscopy testing in series and parallel were also compared against the culture [32]. For this purpose, LAMP and smear microscopy were compared in series (LAMP performed only if smear positive and considered TB positive if both tests are positive or TB negative if either test is negative) and in parallel (LAMP performed for all samples and considered TB positive if either smear or LAMP is positive and TB negative if both are negative). Serial testing improves the specificity but lowers the sensitivity. It also reduces the cost of the second test as it is performed for only those samples positive by the first test. Parallel testing improves the sensitivity, while decreasing the specificity. Cohen's kappa was calculated as a measure of agreement between the tests [33].

### Results and Discussion

The results of the study are outlined in table 1. Samples were considered to be culture negative if no growth was detected in both LJ and MGIT cultures. Samples were considered to be ‘culture positive’ if growth was detected in either LJ or MGIT cultures. Of the 78 samples tested, 7 showed contamination for both LJ and MGIT cultures and were omitted from analysis. 34 samples were LAMP positive, of which one was detected to be contaminated on both LJ and MGIT cultures. Of the 44 LAMP negative samples, 6 were detected to be contaminated on both LJ and MGIT cultures. 33 samples were smear positive. Of the 45 smear negative samples, 7 were detected to be contaminated on both LJ and MGIT cultures. Non-contaminated culture results were available for 33 positive and 38 negative samples each for the LAMP and smear. The performance of LAMP and smear as compared to the LJ or MGIT culture is given in table 2. Efficacy of the LAMP and smear microscopy in series and in parallel is depicted in table 3.

**Table 1:** Results of LAMP, smear and culture.

LAMP	Smear	Number of cultured samples	LJ or MGIT culture			Number of total samples
			Either +	Both -	Both Contaminated	
**+**	**+**	31	31	0	0	**31**
**+**	**−**	2	0	2	1	**3**
**−**	**+**	2	1	1	0	**2**
**−**	**−**	36	7	29	6	**42**
**Total**		**71**	**39**	**32**	**7**	***78***

*Positive results marked as ‘**+**’ and Negative results as ‘**−**‘.

**Table 2:** Three-way comparison of LAMP, smear and culture.

	Culture +	Culture −	Sensitivity (%)	Specificity (%)	PPV (%)	NPV (%)	Positive likelihood ratio	Negative likelihood ratio	k-value
**LAMP +**	31	2	79.5	93.8	93.9	79.0	12.7	0.2	0.7
**LAMP −**	8	30							
n = 71	95% CI =		64.5–89.2	79.9–98.3	80.4–98.3	63.7–88.9	3.3–49.1	0.1–0.4	0.6–0.9
	**Culture +**	**Culture −**							
**Smear +**	32	1	82.1	96.9	97.0	81.6	26.3	0.2	0.8
**Smear −**	7	31							
n = 71	95% CI =		67.3–91.0	84.3–99.5	84.7–99.5	66.6–90.8	3.8–181.7	0.1–0.4	0.6–0.9
	**Smear +**	**Smear −**							
**LAMP +**	31	2	93.9	94.7	93.9	94.7	17.9	0.1	0.9
**LAMP −**	2	36							
n = 71	95% CI =		80.4–98.3	82.7–98.5	80.4–98.3	82.7–98.5	4.6–69.0	0.0–0.2	0.8–1.0
	**Culture +** **Smear +**	**Culture −** **Smear −**							
**LAMP +**	29	0	96.7	100.0	100.0	96.7	NA	0.0	1.0
**LAMP −**	1	29							
n = 59	95% CI =		83.3–99.4	88.3–100.0	88.3–100.0	83.3–99.4	NA	0.0–0.2	0.9–1.0
	**Culture +** **Smear −**	**Culture −** **Smear +**							
**LAMP +**	2	2	22.2	33.3	50.0	12.5	0.3	2.3	NA
**LAMP -**	7	1							
n = 12	95% CI =		6.3–54.7	6.2–79.2	15.0–85.0	2.2–47.1	0.1–1.4	0.5–12.0	NA

**Table 3:** Comparison of LAMP and smear in series and parallel.

		Culture +	Culture −	Sensitivity (%)	Specificity (%)	PPV (%)	NPV (%)	Positive likelihood ratio	Negative likelihood ratio	k-value
LAMP on smear positives	**Positive**	31	0	96.9	100.0					
	**Negative**	1	1							
	(n = 33)	95% CI =		84.3–99.5	20.7–100.0					
2 tests – serial	**Positive**	31	0	79.5	100.0	100.0	80.0	NA	0.2	0.8
	**Negative**	8	32							
	(n = 71)	95% CI =		64.5–89.2	89.3–100.0	89.0–100.0	65.2–89.5	NA	0.1–0.4	0.6–0.9
LAMP on smear negatives	**Positive**	0	2	NA	93.6					
	**Negative**	7	29							
	(n = 38)	95% CI =		NA	79.3–98.2					
2 tests – parallel	**Positive**	32	3	82.1	90.6	91.4	80.6	8.8	0.2	0.7
	**Negative**	7	29							
	(n = 71)	95% CI =		67.3–91.0	75.8–96.8	77.6–97.0	65.0–90.2	3.0–26.0	0.1–0.4	0.6–0.9

LAMP detects the presence/absence of the genetic material of *M. tuberculosis*. Smear microscopy detects the morphology and culture methods differentiate based on the physiology of the viable organism. These different approaches could be complementary. In this study, the LAMP was observed to have high sensitivity and specificity for samples with concordant culture and smear results. The overall efficacy of LAMP and fluorescence smear microscopy in the current study was high and broadly similar. However, the performance of LAMP in smear negative samples was found to be suboptimal.

The LAMP showed excellent sensitivity (96.7%) and specificity (100.0%) for those samples with analogous culture and smear results (n = 59), as shown in table 2. These values are similar to those reported earlier [24]. LAMP showed poor sensitivity (22.2%) and specificity (33.3%) for the samples with discordant culture and smear results (n = 12, Table 2). The overall sensitivity (79.5%) and specificity (93.8%) of LAMP was slightly lower than that of the smear microscopy (82.1% and 96.9% respectively) for the entire samples (n = 71) when compared to culture. LAMP showed a sensitivity and specificity of 96.9% and 100.0% respectively for smear positive samples (n = 33). The specificity of LAMP for smear negative samples (n = 38) was 93.6%. The sensitivity of LAMP for smear negatives could not be calculated due to the limited number of samples. When both the LAMP and smear microscopy were performed serially, the overall sensitivity and specificity observed were 79.5% and 100.0% respectively (n = 71). When both the LAMP and smear microscopy were performed in parallel (n = 71), the sensitivity and specificity observed were 82.1% and 90.6% respectively (Table 3). Commercial NAATs had shown pooled sensitivity and specificity of 96.0% and 85.0% respectively among smear positive samples and 66.0% and 98.0% respectively among smear negative samples[34]. In-house PCR assays were reported to show pooled sensitivity and specificity of 96.0% and 81.0% respectively for smear positive samples [35], indicating that a standardized LAMP can perform potentially better than or equivalent to PCR-based methods.

LAMP does not appear to pick up any additional true positives or pick up any that the smear missed, in smear negative samples. Hence the LAMP assay in the current format may be useful only in diagnosis of smear positive samples or as a rule-in test for smear negative samples (specificity of 93.6%). The utility of LAMP as a tool to resolve differences between the culture and smear results is questionable, especially when smear negative samples are involved. However, this has to be validated with larger sample sets.

Discordant results between the LAMP and smear or culture can arise if non-tuberculous Mycobacteria (NTM) are present [36], [37] or if cultures are contaminated [38]. NTM have been reported at high frequencies from southern India [39], [40]. The development of a LAMP assay capable of identifying both MTBC and NTM can improve the sensitivity and specificity significantly. In this study, the one sample that was LAMP negative but smear and LJ positive was later identified as MTB by the ‘GenoType MTBC’ assay (HAIN lifesciences GmbH, Germany) as well as by LAMP for culture lysates. These rule out NTM or sequence variation as reasons for amplification failure with this sample and indicate the lower concentration of nucleic acid present or the presence of inhibitors. All samples negative by LAMP were spiked with *M. tuberculosis* DNA and re-amplified. As all these samples amplified, presence of inhibitors can be ruled out.

The high sensitivity observed for smear microscopy in this study may be due to the nature of sampling, better lab practices or the high prevalence of TB as observed elsewhere [41], [42]. One of the factors reducing the sensitivity of the LAMP may be the lysis of specimens upon storage [43]. Subsequent supernatant removal and NaOH treatment of lysed specimens may reduce the amount of amplifiable DNA available. Washing and NaOH-free methods for sputum processing will be of considerable value to NAATs.

### Conclusions

LAMP appears to be suitable for smear positive specimens with concordant culture results, but was ineffective in smear negative samples. LAMP and smear in series had high specificity (100.0%) and can be used as a rule-in test combination. An improved LAMP alone or together with smear microscopy can offer same-day diagnosis and has the potential to reduce the drop-out rate substantially. However, these findings need to be substantiated further with larger sample sizes and in different geographical settings. Inter-reader reproducibility and the performance with smear negative samples also need further evaluation. Suitably modified, LAMP can potentially improve the diagnosis of TB in resource limited settings.

### Limitations

In the current study, sample size under certain subtypes was too limited to arrive at statistically significant estimates of efficacy. The sampling was not continuous due to limited availability of stored samples. Speciation of all the cultured samples were not performed and as such it is not possible to determine how many of the culture or smear positives were NTM, which the LAMP will not detect using current set of primers. The HIV status was not ascertained for the study samples. However, it is unlikely that HIV had a significant impact on the findings, as other studies in the same region indicate <1% prevalence for HIV.
